# New Insights into the Phylogeny of the A.Br.161 (“A.Br.Heroin”) Clade of *Bacillus anthracis*

**DOI:** 10.3390/pathogens13070593

**Published:** 2024-07-16

**Authors:** Markus Antwerpen, Wolfgang Beyer, Gregor Grass

**Affiliations:** 1 Bundeswehr Institute of Microbiology (IMB), 80937 Munich, Germany; 2 Department of Livestock Infectiology and Environmental Hygiene, Institute of Animal Science, University of Hohenheim, 70599 Stuttgart, Germany

**Keywords:** *Bacillus anthracis*, anthrax, phylogenetics, PCR assay, heroin

## Abstract

*Bacillus anthracis* is a rare but highly dangerous zoonotic bacterial pathogen. At the beginning of this century, a new manifestation of the disease, injectional anthrax, emerged as a result of recreational heroin consumption involving contaminated drugs. The organisms associated with this 13-year-lasting outbreak event in European drug consumers were all grouped into the canonical single-nucleotide polymorphism (canSNP) clade A-branch (A.Br.) 161 of *B. anthracis*. Related clade A.Br.161 strains of *B. anthracis* not associated with heroin consumption have also been identified from different countries, mostly in Asia. Because of inadvertent spread by anthropogenic activities, other strains of this A.Br.161 lineage were, however, isolated from several countries. Thus, without additional isolates from this clade, its origin of evolution or its autochthonous region remains obscure. Here, we genomically characterized six new A.Br.161 group isolates, some of which were from Iran, with others likely historically introduced into Germany. All the chromosomes of these isolates could be grouped into a distinct sub-clade within the A.Br.161 clade. This sub-clade is separated from the main A.Br.161 lineage by a single SNP. We have developed this SNP into a PCR assay facilitating the future attribution of strains to this group.

## 1. Introduction

The zoonotic disease anthrax that is caused by the bacterium *Bacillus anthracis* exhibits three classical manifestations in humans [1]. The most severe is inhalation (pulmonary) anthrax with case fatality rates approaching 100% if left untreated and remains high even with antibiotic therapy. Intestinal anthrax is also a severe infection but does not quite reach the mortality of inhalation anthrax. Both these manifestations are rare, whereas cutaneous anthrax, the least lethal infection route, is the most prominent one, in about 95% of all cases [1]. The year 2000 saw a fourth kind of manifestation of *B. anthracis* infection emerging. A recreational heroin consumer suffered a severe soft-tissue infection at the drug injection site. The patient eventually experienced cardiovascular shock and died of anthrax meningitis [2], a rare but typical complication of anthrax infection [3]. More cases of injectional anthrax followed from 2009 until 2013 [4], with up to 141 further patients conducting the disease (including likely and presumed cases) with an overall case fatality rate of 35% [5].

Though *B. anthracis* was isolated from European injectional anthrax patients in 2000, 2008–2009, and 2012–2013, these isolates were genotypically all very similar [4,6,7,8]. Finally, a study compiling genomic sequences of all 60 then-available isolates revealed that these strains share a very similar genomic makeup [9]. Only a small number (37) of single nucleotide polymorphisms (SNPs) separate the genomes of these isolates and just 16 SNPs divide two tightly nested clusters with most isolates being SNP identical or showing only one or two unique SNPs [9]. The canonical genotype to which all injectional anthrax isolates belong, i.e., canSNP clade A.Br.161 (also known as “A.Br. Heroin” [10]), was uncommon for the European countries from which the infected patients originated [6,9]. Instead, only a few related clade A.Br.161 strains were found in collections and these isolates had no known connection to heroin. These strains had been isolated earlier from diverse countries such as Iran, Turkey, and the United States of America [9,10,11]. In the latter case, the isolates likely derived from imported goods into the USA (e.g., via tanneries processing contaminated animal hides, or wool) [9].

Though the true origin of the heroin-associated *B. anthracis* strains still remains unknown [9], the canSNP group to which they belong is worthy of continued interest. Recently, Shevtsov and co-workers have analyzed A.Br.161 isolates from Kazakhstan [12]. The authors noticed these strains were isolated from certain regions of Kazakhstan (Turkistan, South Kazakhstan) which lay along the ancient central Asian terrestrial trade route network collectively termed “Silk Roads”. If there is a strong connection between these trade routes and the A.Br.161 clade, we can expect a wide dispersal of this branch of *B. anthracis* not only in West and Central Asia but also across diverse parts of the world.

It is very likely, that, with additional strains added to this group, more detailed information can be gathered concerning the phylogeny of this prominent lineage of the anthrax pathogen. Thus, here we have genotyped and genome-sequenced six additional isolates from the A.Br.161 canSNP clade and positioned their chromosomes within this phylogenetic group. All six new strains can be grouped into a single sub-clade. This sub-clade comprises (comparatively) basal group members, which are relatively distantly related to the heroin-associated outbreak strains that have infected European heroin consumers.

## 2. Materials and Methods

### 2.1. Bacterial Culture and Inactivation and Genomic DNA Preparation

*B. anthracis* strains (Table S1) were grown on Columbia blood agar (Becton Dickinson, Heidelberg, Germany) or trimethoprim/sulfamethoxazole/polymyxin blood agar (TSPBA) [1,13], and cultures were chemically inactivated with 4% (*v*/*v*) Terralin PAA (Schülke & Mayr GmbH, Norderstedt, Germany), as in [14]. Genomic DNA was isolated using the MasterPure Gram Positive DNA Purification kit (LGC, Teddington, UK) as described for Gram-positive bacteria. DNA concentrations were quantified using the Qubit dsDNA HS Assay kit (Thermo Fisher Scientific, Dreieich, Germany) according to the supplier’s protocol. DNA preparations were stored at −20 °C until further use.

### 2.2. Interrogation of canSNPs via PCR by Relative Ct-Value Analysis (Delayed Mismatch Amplification PCR Assay, DMAA)

Delayed Mismatch Amplification PCR Assays (DMAAs) were used to determine clade-specific canSNPs in genomic *B. anthracis* DNA [11]. This probe-free, real-time qPCR assay is based on the ‘Melt analysis of mismatch amplification mutation assays’ (melt-MAMAs) technique [15]. Both techniques interrogate the character state of SNPs (derived, DER, vs. ancestral, ANC) in two separate PCR reactions containing matching or mismatching forward primer oligonucleotides preferentially favoring amplification of one of the two alleles (DER or ANC, respectively) and a shared reverse primer. Subsequent analysis of the PCR runs is accomplished by a simple relative subtraction of numerical PCR threshold values (Ct values; ΔCt = Ct(DER) − Ct(ANC)) [11]. *B. anthracis* Sterne DNA served as an ancestral DNA of strain UR-1 [16] or 3016 [11] as derived state references, respectively, depending on the assay. DMAA-SNP primer sequences for real-time PCR assays are listed in Table S2. Each primer pair was used in a 20 μL single-plex reaction. For this, 1 μM of each primer pair and approximately 20 ng of the template DNA were added to 1× LightCycler 480 SYBR Green I Mastermix (Roche, Mannheim, Germany). The amplification and recording of Ct values was carried out on a LightCycler 480 II instrument (Roche, Mannheim, Germany) as described in [17], without melting curve analysis.

### 2.3. Whole-Genome Sequencing

For obtaining whole-genome sequences of *B. anthracis* strains, sequence libraries were prepared using the NEBNext^®^ Ultra™ II DNA Library Prep kit (New England Biolabs, Frankfurt am Main, Germany) or Illumina DNA Prep (Illumina, Berlin, Germany) with 100 ng of input DNA. The subsequent use of the Illumina MiSeq platform with 2 × 300 bp v3 chemistry produced at least 1,206,811 reads for each isolate (IP-4001,IP-4009, A033 and A034). For the remaining samples, A162 and A164, the NextSeq 2000 system was used with 2 × 150 bp P2 chemistry producing at least 2,545,254 reads for each sample.

All sequence reads were assembled *de novo* using an in-house script based on the SPAdes (version 3.15.3) assembler [18] to create draft genomes. For correcting SNPs or closing small gaps and INDELs, Pilon (version 1.24) [19] was used. For these polished scaffolds, the *B. anthracis* canSNP group was determined using canSNPer software Version: v1.0.10 (10.1093/bioinformatics/btu113, accessed on 30 January 2024) in combination with the canSNPer2 database (https://github.com/FOI-Bioinformatics/CanSNPer2-data, accessed on 30 January 2024). All data generated or analyzed during this study are included in this published article and their supplementary information is publicly available in the NCBI Sequence Read Archive (SRA) repository; Bioproject PRJNA309927. The obtained scaffolds were manually checked for contaminant reads and annotated automatically by the NCBI Prokaryotic Genome Annotation Pipeline [20] after submission.

### 2.4. Analysis of Whole-Genome Sequencing Data and SNP Calling

Prior to core chromosome multiple-sequence alignment with SNP calling using the Parsnp tool from the Harvest Suite (version 1.1.2) [21], representative genomes and scaffolds of *B. anthracis* belonging to the A.Br.161 canSNP group were downloaded from the ncbi SRA database or genome repository and assembled as described above if needed. All of these chromosomes were aligned using Parsnp (parameters-c-e-u-C 1000) against the reference chromosome of *B. anthracis* ‘Ames Ancestor’ (NC_007530) which also served as a phylogenetic outgroup. For further analysis of the identified SNP positions, HarvestTools (version 1.2) of the same software suite [21] was used to export these SNP positions and create a variant calling file (vcf). In order to avoid biased estimations and enhance data quality, chromosome regions with closely adjacent SNPs (<10 bp distance) and positions harboring undefined nucleotides (“N”) were masked for further analyses. Using this curated vcf file (Table S3), a multi-FASTA file was compiled from the chromosome dataset comprising the concatenated 1550 SNPs as a multiple-sequence alignment. This concatenated sequence information was finally used to calculate a set of the most parsimonious trees using the parsimony ratchet [22] with nearest neighbor interchange rearrangement via software R Version: 4.3.0 in combination with package Phangorn [23]. From this, a Newick tree was exported and loaded into the Grapetree tool [24] for visualization, and subsequently manually edited (using PowerPoint 2016, Microsoft, Munich, Germany) for optimization of the style.

## 3. Results and Discussion

### 3.1. Identification of Additional Isolates of canSNP Clade A.Br.161 from Strain Collection

Phylogenetic information on the A.Br.161 clade is currently heavily biased towards the plethora of isolates recovered from the heroin-associated outbreak of injectional anthrax in Europe [9]. In order to gain more insight into this clade’s diversity, we initially screened genomic DNA of canSNP A.Br.008/009 *B. anthracis* isolates of our strain collection by DMAA-PCR. These isolates had not yet been further interrogated for derived states of canSNP A.Br.161. We interrogated for the clade-specific SNP at position 5,013,862 in the reference chromosome NC_007530. This is one of the SNP positions that define this clade [10]. From this analysis, six additional A.Br.161 isolates, A033, A034, A162, A164, IP4001, and IP4009 (Table S1), were identified that featured a derived SNP state (Table 1). Strains A033, A034, A162, and A164 were all possibly collected in 1957 by a veterinary diagnostic center in southern Germany (Table S1). There is little information available besides the isolation year and the fact that these strains were likely collected from an outbreak in animals. These strains represent thus the first likely animal-derived, non-heroin-associated A.Br.161 strains identified in Germany or anywhere else from Western Europe. However, it is very likely that these strains have originally been imported (as were the heroin-associated strains), e.g., as contaminants of animal products such as hides processed at a tannery [25]. Outbreaks in grazing animals like bovines and sheep around tanneries were not uncommon in past times well into the 20th century. *B. anthracis* spores can still be found around such localities as they may persist in a viable state in soil for many years, likely even decades [26,27]. Moreover, the original spores at aging contaminated sites might encounter temporally favorable conditions for germination and limited reproduction [17]. Besides the proliferation *of B. anthracis* within the plant rhizosphere [28], there might be a possibility that the local spore pool is maintained by sporadic germination, growth, and sporulation by passages through amoeba [29] or invertebrates such as earthworms [30]. Such intermittent reproductive cycles may thus extend the life expectancy of the local spore pool and may thus constitute the source of animal infection [17,31]. Two further isolates identified by us (Table 1) as belonging to the A.Br.161 clade (IP4001 and IP4009) had been isolated in Iran in 1936 or 1996, respectively (Table S1). No further information is available for these strains.

### 3.2. New Genomes Complement the Phylogeny of the A.Br.161 canSNP Clade

**Figure 1:**
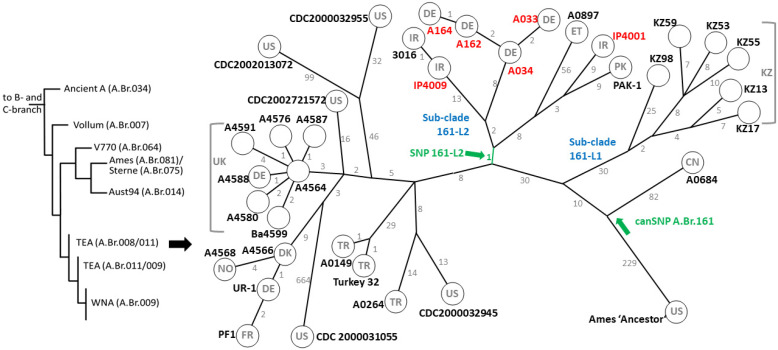
Phylogenetic position and minimum spanning tree (MST) of canonical A.Br.161 clade of *B. anthracis*. (**left**) Overview of phylogenetic placement of canSNP clade A. Br.161 within the global diversity of *B. anthracis*; (**right**) A MST was calculated from 1550 chromosomal SNPs and visualized using Grapetree [24]. Numerical SNP distances between chromosomes (respective strains are named in bold-type letters) are indicated at branches. Newly sequenced isolate names are displayed in red, sub-clades in blue, and relevant SNP positions in green characters. Countries from which respective strains were isolated are ISO 3166-1 alpha-2 coded as grey letters: CN—China, DE—Germany, DK—Denmark, ET—Ethiopia, FR—France, IR—Iran, KZ—Kazakhstan, NO—Norway, PK—Pakistan, TR—Turkey, UK—United Kingdom, US—United States of America.

Next, we sequenced the genomic DNA of the SNP-identified isolates and mapped the chromosomal reads to the *B. anthracis* Ames ‘Ancestor’ reference chromosome. Analysis of these and of 28 additional chromosomal sequences from public databases belonging to the A.Br.161 clade yielded a total of 1550 chromosomal SNP positions (Table S3). Figure 1 illustrates the position of the A.Br.161 clade within the global phylogeny of *B. anthracis* and the new strains within a minimum spanning tree (MST) tree calculated from the chromosomal SNP data.

The root, the Ames ‘Ancestor’ chromosome was at a 229 SNP distance from the basal node of the A.Br.161 clade (Figure 1). The most basal strain, A0684, of the A.Br.161 clade featured 82 autapomorphous (i.e., isolate-specific, derived characters) SNPs from the node of this single-isolate branch. Isolate A0684 originated from China [10], which fits well with the proposed dispersal of spores of this clade along the Silk Roads of Persia and Central and East Asia [12]. Supporting this model is the placement of the next sub-clade along the A.Br.161 branch. This sub-clade, named here 161-L1, is currently made out of six strains and is entirely composed of isolates from Kazakhstan [12], a country well embedded in the Silk Roads network. Also, this sub-clade is defined by 30 synapomorphous (i.e., shared and clade-specific, derived characters) SNPs. The minimum and the maximum internal SNP distance between any two chromosomes was 12 and 45 SNPs, respectively. Clearly, based on their SNP distances, the isolates of this sub-linage represent short yet individual evolutionary paths rather than a single common outbreak origin [32]. Re-markably, Afghanistan, the country which is responsible for about 90% of the global heroin production, is probably not contributing to the A.Br.161 clade diversity. As re-ported previously, 17 Afghan animal isolates of B. anthracis group into the distinct A.Br.Vollum (A.Br.007) clade [7] (Fig. 1, left panel). Up until now, to the best of our knowledge, no additional *B. anthracis* genomes have been reported from this country. Thus, there is no immediate connection between Afghanistan and the A.Br.161 clade besides heroin trafficking and the historic Silk Roads network.

The following sub-clade, named here 161-L2, featured a different topology (Figure 1). This is also the sub-clade into which every newly sequenced genome could be placed. Notably, there was a single synapomorphous SNP at the basis of this group of isolates after which two branches diverged. Herein, the minimum SNP distance between any two chromosomes was one, and the maximum was 80 SNPs. However, there were chromosomes that differed only by a single SNP from their closest neighbors (A162, A164, and 3016, IP4009, respectively). This strongly suggests that these very close relatives may either represent basically identical strains or different isolates from single outbreaks. In the case of A033, A034, A162, and A164, the odds are that all these isolates represent individual isolates from a single bovine anthrax outbreak in southern Germany. This notion is supported by the maximum differences between these chromosomes of five SNPs. This number is well in the range of SNPs defining a single (broader) outbreak event as stated earlier [32]. Thus, similar to heroin-associated *B. anthracis*, all these newly characterized strains attributed to the A.Br.161 clade have very probably been imported from distant world regions to Europe. This finding is thus no strong argument against the evolution of the A.Br.161 canSNP clade in Asia, especially when other members of the SNP 161-L2 sub-clade are clearly supporting the Asian origin of the A.Br.161 canSNP clade.

Nowadays, the contamination of hides and wool with *B. anthracis* spores is no longer an issue because in most countries import of animal products has become regulated [1]. Up to well into the last century, however, spore contamination was the rule rather than the exception. This also explains the term “Wool Sorter’s Disease” as a trivial name for anthrax [1]. Possibly, the placement of another strain (A0897) within this 161-L2 sub-clade can be explained likewise through human activities, even though no supporting metadata for this isolate were available at the time of this writing. Strain A0897 was initially described as an equine isolate from Ethiopia [7]. It can be anticipated that we will only gain further insight into the relevance of this geographic outlier once more isolates from this African country have become genomically characterized. Conversely, the two remaining isolates of this sub-clade were isolated from Iran (IP4001) or Pakistan (PAK-1). Their origin again supports the idea of an Asian origin of the canSNP clade A.Br.161. Still, mindful of the position of both basal sub-clade 161-L1 and the unique most basal isolate A0684, the birthplace of the A.Br.161 lineage is most possibly located further east than Turkey, Iran, or even Pakistan (Figure 1).

The remainder of canSNP clade A.Br.161 comprised previously characterized chromosomes [9]. These were the closer relatives of the heroin consumption-associated strains and the actual isolates from patients suffering from injectional anthrax [9]. As noted previously, this part of the A.Br.161 lineage includes outbreak strains from Turkey (A0264, A0149, and Turkey32) [10,27], historic isolates from the USA of diverse but in all likelihood of imported origin [10], and the *sensu stricto* heroin consumption-associated isolates from several European countries [9]. Among these, there is the chromosome of the isolate (A4568) representing the first ever reported case of injectional anthrax from a heroin “skin popper” (i.e., the drug was injected subcutaneously, not intravenously) in Norway from 2000 [2]. Intriguingly, this first injectional anthrax isolate from the year 2000 was among the most distant chromosomes from the polytomy at the base of the *sensu stricto* heroin-associated isolates. Of note, (disregarding the outlier isolate CDC2000031055 [10]) the greatest distance within the *sensu stricto* heroin group was 23 SNPs, while the distance from the polytomy at the base of the *sensu stricto* heroin-associated isolates to the branching point of the most basal A.Br.161 strain A0684 accumulated to 55 SNPs (Figure 1). This clearly correlates with the relatively young evolutionary age of the *sensu stricto* heroin-associated sub-clade especially in relation to the 161-L2 sub-clade comprising all of the newly sequenced chromosomes (only 15 SNPs from the 161-L2 base node to the polytomy of the *sensu stricto* heroin-associated isolates) (Figure 1).

### 3.3. A New PCR Assay for the Facile Interrogation of an Informative SNP Position of the canSNP A.Br.161 Sub-Clade 161-L2.

SNP information from Table S3 visualized in Figure 1 prompted us to design and test a new DMAA PCR assay. There was a single SNP position at the base of sub-lineage 161-L2 (at position 906,760 in the *B. anthracis* ‘Ames Ancestor’ reference chromosome, NC_007530) that separated this lineage from the rest of the A.Br.161 phylogeny. Thus, this unique branching point can be interrogated by PCR. Indeed, the DNA of all seven 161-L2 lineage isolates available to us featured a derived allele genotype when DMAA-PCR assayed (Table 1). The DNA of Sterne (A.Br.075 canSNP group) and the injectional anthrax isolate UR-1 featured, as expected, the ancestral allele genotype for the 161-L2 sub-clade.

The DNAs of *B. anthracis* isolates from injectional anthrax victims have been genotyped by melt-MAMA PCR before [15]. DMAA-PCR [11] is a direct continuation of the earlier melt-MAMA-PCR technique. Melt-MAMA makes use of the melt-curve analysis at the end of the PCR reaction (alternatively, analysis can be performed by separating amplicons on agarose gels, with one of the two allele reactions employing GC-clamp base extensions for size differentiation) [15,33]. DMAA-PCR facilitates analysis by simply comparing the threshold values (Ct) of the derived vs. ancestral primer reactions run in parallel [11]. As such, a DMAA is easier to implement than melt-MAMA, which can be limited by SNPs representing a transition or by structural constraints of the primers [15]. Of note, the template DNA concentration is variable as long as parallel DER/ANC-PCRs are run with equal DNA amounts (Table 1). In contrast to melt-MAMA, however, which can be implemented as agarose-gel-based assays [15,33], a DMAA is strictly dependent on real-time PCR capabilities. Typically, we achieve a >90% success rate in new DMAAs without optimization compared to about 80% in melt-MAMA after optimization as published earlier [15]. Together with the previously published PCR typing assays (published as melt-MAMA analysis) [9], the two new DMAA-PCR assays for canSNP A.Br.161 and the sub-clade SNP 161-L2 should make it straightforward to quickly genotype new A.Br.161 isolates as soon as these become available.

## 4. Conclusions

As suggested previously [12], our results strengthen the view of a strong connection between the historic trade routes (Silk Roads) and the likely natural habitat of the A.Br.161 clade, i.e., the Middle East and Central Asia. The occurrence of members of this clade in other parts of the world is thus likely a result of the anthropogenic dispersal of this branch of *B. anthracis* not only historically (e.g., via Silk Roads and sea trade) but also due to the recent inadvertent co-transport with heroin.

## Figures and Tables

**Table 1:** DMAA PCR-based SNP typing of *B. anthracis* strains.

Strain	Ct Range (Derived Allele, DER)	Ct Range (Ancestral Allele, ANC)	Δ(Ct_DER_ − Ct_ANC_)	SNP Allele	SNP Group ^1^
Sterne	38.7–40.0	28.7–28.8	10.6 ± 0.9	ANC	Non-A.Br.161
UR-1	23.1–23.7	33.9–34.1	−10.6 ± 0.3	DER	A.Br.161
3016	25.7–26.0	39.1–40.0	−13.7 ± 0.5	DER	A.Br.161
IP4009	26.5–26.8	39.2–40.0	−13.4 ± 0.2	DER	A.Br.161
IP4001	26.3–26.8	37.0–40.0	−15.4 ± 3.2	DER	A.Br.161
A033	20.9–22.2	32.2–34.1	−11.7 ± 0.3	DER	A.Br.161
A034	20.0–21.5	33.8–35.6	−13.9 ± 0.2	DER	A.Br.161
A162	21.0–21.5	37.4–40.0	−17.5 ± 1.4	DER	A.Br.161
A164	20.1–20.2	35.5–37.0	−16.0 ± 1.1	DER	A.Br.161
Sterne	38.4–38.5	27.7–27.9	10.7 ± 0.1	ANC	Non-161-L2
UR-1	28.8–32.2	16.0–19.8	12.6 ± 0.4	ANC	Non-161-L2
3016	19.5–23.1	30.8–34.9	−11.5 ± 0.3	DER	161-L2
IP4009	18.5–22.8	30.9–35.5	−12.5 ± 0.2	DER	161-L2
IP4001	18.8–22.9	30.8–35.3	−12.2 ± 0.3	DER	161-L2
A033	18.0–18.5	30.0–30.1	−11.8 ± 0.4	DER	161-L2
A034	17.2–17.3	28.8–28.9	−11.7 ± 0.2	DER	161-L2
A162	14.2–17.4	25.9–29.6	−11.9 ± 0.4	DER	161-L2
A164	13.8–16.6	26.2–28.8	−12.3 ± 0.2	DER	161-L2

^1^ SNP A.Br.161: previously defined SNP at position 5,013,862 [10] (top); and new A.Br.161 sub-clade SNP 161-L2 at position 906,760 (bottom) in reference chromosome NC_007530.

## Data Availability

The genome sequence data presented in this study are available from the NCBI database under the BioProject ID: PRJNA309927. These and the accession numbers of publicly available genome sequences analyzed are listed in the Supplementary Materials (Table S1) of this study.

## Supplementary Materials

The following supporting information can be downloaded at: https://www.mdpi.com/article/10.3390/pathogens13070593/s1, Table S1: *Bacillus anthracis* strains analyzed in this study; Table S2: Primers used for DMAA analysis; Table S3: SNP matrix of analyzed *Bacillus anthracis* chromosomes.
